# Optical Refractive Index Sensing Based on High-*Q* Bound States in the Continuum in Free-Space Coupled Photonic Crystal Slabs

**DOI:** 10.3390/s17081861

**Published:** 2017-08-11

**Authors:** Yonghao Liu, Weidong Zhou, Yuze Sun

**Affiliations:** Department of Electrical Engineering, University of Texas at Arlington, Arlington, TX 76019, USA; yonghao.liu@mavs.uta.edu

**Keywords:** optical sensor, Fano resonance, label-free sensor, photonic crystals, bound states in the continuum

## Abstract

High sensitivity (*S*) and high quality factor (*Q*) are desirable to achieve low detection limit in label-free optical sensors. In this paper, we theoretically demonstrate that single-layer and coupled bi-layer photonic crystal slabs (PCS) possess simultaneously high *S* and high *Q* near the bound states in the continuum (BIC). We theoretically achieved *S* > 800 nm/RIU and *Q* > 10^7^ in refractive index sensing in the 1400–1600 nm telecom optical wavelength bands. We experimentally demonstrated an *S* of 94 nm/RIU and a *Q* of 1.2 × 10^4^, with a detection limit of 6 × 10^−5^ refractive index unit. These sensor designs can find applications in biochemical sensing, environmental monitoring, and healthcare.

## 1. Introduction

Optical biosensors are widely-used for biomedical research, healthcare, and environmental monitoring. Compared with optical sensors based on fluorescence measurement, label-free optical sensors do not need to label the biomolecules, which reduces the effort for sample preparation and enables rapid analysis in real time [1,2]. Refractive index (RI) detection is commonly used for label-free sensors. RI-based sensing has been studied in the analysis of biochemical molecules detection [3,4,5,6,7], drug compound screening [8], and gas detection [9,10,11].

The performance of a sensor can be fully described by detection limit (*DL*). High quality factors (*Q*) or RI sensitivities (*S*) for the resonance will help to achieve a lower detection limit [1]. Surface plasmon resonance (SPR) biosensors have been widely studied for localized RI sensing [12,13], and they are commercially available. SPR biosensors can provide high sensitivity *S* but the large absorption in metal leads to low *Q*. Photonic crystal (PhC) devices, such as localized PhC defect cavities [3,14,15,16], slotted PhC cavities [17,18,19,20], and free-space coupled defect-free photonic crystal slabs (PCS) [4,7,21,22,23,24,25], have been widely explored for RI sensing to build compact on-chip integrated sensors with high performance. Localized PhC defect cavities offer a moderately high *Q* of 10^4^ to 10^5^, but small overlap of the field with the analyte limits the sensitivity to around 100 nm/RIU [3,14,15,16]. Slotted PhC cavities can achieve higher sensitivity by increasing the mode field overlap with the analyte [20]. However, the device is sensitive to fabrication imperfections, which limits the ultimate quality factors that can be achieved in practice. High *Q* of 10^7^ is possible to achieve theoretically in slotted PhC cavities [19], while a *Q* of 10^3^ to 10^4^ has been demonstrated experimentally [17,18,20]. In order to couple the light from fiber into the on-chip waveguide for localized defect and slotted PhC cavities, delicate alignment is typically required to reduce the coupling loss. Additionally, sensor response time could be negatively impacted by the slow diffusion of analytes to the sub-micrometer sized defect cavities and slot region.

Fano resonances in the PCS structures could be excited efficiently by the external light that is vertically incident on the PCS [26,27,28,29]. A bound state in the continuum (BIC) are waves that coexist with the continuous radiation spectrum, remaining perfectly confined without any radiation [30,31]. BICs exist in one dimensional (1D) and two dimensional (2D) photonic crystals at wave vector ***k*** ≈ 0 and some discrete ***k*** points with infinite lifetime and infinite *Q* [32,33,34,35]. Fabry-Perot BICs can be formed in coupled bi-layer PCS by choosing an appropriate spacing between the two slabs [30,35,36]. An extremely high *Q* can be achieved when coupling to these perturbed BICs by tilting the light away from the surface-normal direction [26,32,33], or tuning the spacing between coupled bi-layer PCS [35,36,37]. In a previous study, we demonstrated experimental result of RI sensing with high-*Q* BIC at ***k*** ≈ 0 [22], with a quality factor of 3.2 × 10^4^ and a low detection limit ~3 × 10^−5^ RIU.

In this work, we propose single-layer and coupled bi-layer PCS structures to achieve simultaneous high *Q* and high *S* based on BIC in free-space coupled PCS. By engineering the parameters of the PCS and the light incident angle, the theoretical *Q* of the resonance is tunable in a wide range from a few hundred to infinity. We study the high *Q* condition in each proposed device structure and discuss several designs that are easy to maintain extremely high *Q* (>10^7^), taking into consideration the experimental implementation challenges. The designs presented in this work are promising platforms to achieve extremely low detection limits for RI sensing in practice.

## 2. Device Structure and Computational Methods

**Figure 1:**
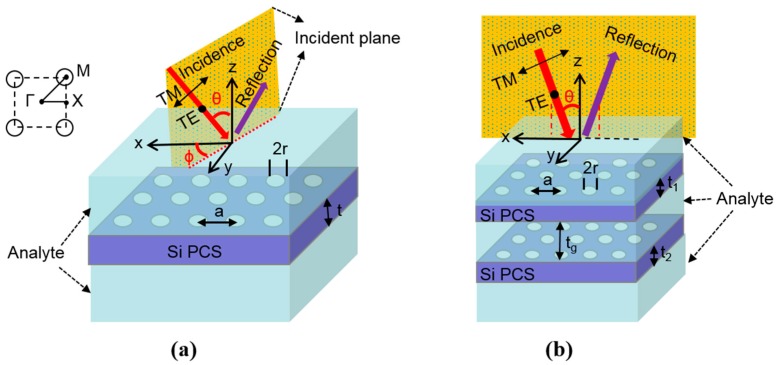
Schematic and key design parameters of the proposed sensor structures, with angle and polarization defined for incident light beam: (**a**) Single layer PCS, where *a* is the lattice constant, *r* is the hole radius, *t* is the Si PCS thickness, *θ* is the incident angle, and *φ* is the azimuth angle. The inset shows the Brillouin zone symmetric points in the ***k***-space (Γ, X and M) for the photonic crystal lattice; and (**b**) Coupled bi-layer PCS where *t*_1_ is the top Si PCS thickness, *t*_2_ is the bottom Si PCS thickness, and *t_g_* is the air gap distance between the top and the bottom Si PCS.

Figure 1a shows the schematic of a single-layer Fano resonance square lattice PCS sensor, where key design parameters are defined as lattice period (*a*), air hole radius (*r*), and thicknesses of the Si PCS (*t*). As shown in Figure 1a, two angles are defined to represent the propagation direction of the incident beam, the incident angle *θ* (the angle between incident beam and the surface-normal *z* axis) and the azimuth angle *φ* (the angle between the positive *x*-axis and the incident beam projection in the *x*–*y* plane). The Si PCS is immersed in symmetric aqueous analyte (RI = 1.33), which also fill the air holes. The refractive index of Si is taken as 3.48. A portion of the incident light gets reflected back and the other portion of light gets transmitted through the PCS, neglecting the absorption in the analyte and the PCS. The second configuration consists of two Si PCS separated with an air gap distance *t_g_*, as shown in Figure 1b. The thickness of the top Si PCS is *t*_1_ and the bottom Si PCS is *t*_2_. The coupled PCS is immersed in analyte with the air hole and air gap filled with analyte. 

Reflection spectra of the PCS are simulated with a Fourier Modal Method (FMM) based software package Stanford Stratified Structure Solver (S^4^) [38]. The simulation of angular dependence of the modes is similar to our previous work [39,40]. One unit cell of the PCS is simulated in S^4^. Spectral sensitivity (∆*λ*/∆*n*_analyte_) is calculated by tracking Fano resonance shift for the PCS structure with a different RI of analyte. The quality factor is calculated by performing Fano fitting of the simulated spectra [41]. The polarization of the incident beam is defined with respect to the incident plane, as shown in Figure 1. The transverse electric (TE) polarized beam has an electric field perpendicular to the incident plane, and the transverse magnetic (TM) polarized beam has an electric field parallel to the incident plane.

Field distribution is computed with a finite-difference time-domain (FDTD) based software MEEP [42]. A unit cell of the PCS was simulated with excitation of a planar Gaussian source. Small changes in the RI of the analyte in the vicinity of the PCS will cause a spectral shift of the Fano resonances, with a relation of first-order approximation between the spectral shift and the RI change based on perturbation theory [43]. The optical overlap integral *f* can be defined as the ratio of electric field energy in the analyte region to the total energy for a given mode [24].

(1) $$f=\frac{\int_{V_{analyte}}\varepsilon {\left|E\right|}^{2}dv}{\int_{V_{total}}\varepsilon {\left|E\right|}^{2}dv}$$

where *ε* is the material dielectric constant, and *E* is the electric field. The bulk spectral sensitivity *S* and the optical overlap of integral *f* can be related through the following equation

(2) $$S=\frac{\Delta \lambda_{0}}{\Delta n_{analyte}}=\lambda_{0}\frac{f}{n_{analyte}}$$

where *λ*_0_ is the resonance wavelength and *n_analyte_* is the refractive index of the analyte. We calculated *f* from the field distribution with MEEP based on Equation (1) and then derive the sensitivity from Equation (2), which is consistent with the result calculated from the resonance spectral tracking using S^4^. 

## 3. Simulation Results 

### 3.1. Single Layer PCS

#### 3.1.1. Case S_1_: Surface-Normal Incidence

**Figure 2:**
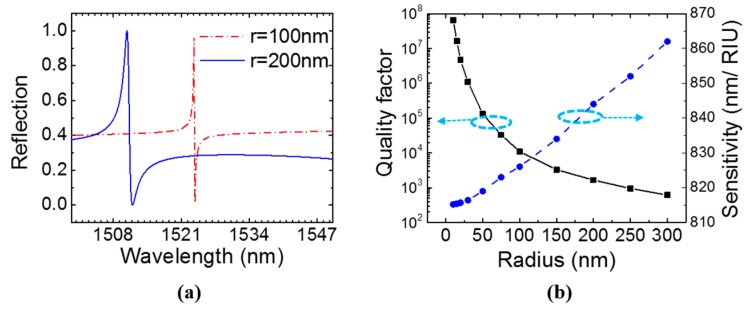
Simulation of single-layer PCS with *a* = 1000 nm and *t* = 160 nm at surface-normal incidence (*θ* = 0°). (**a**) Reflection spectra of PCS with two different hole radiuses. (**b**) Quality factor and sensitivity of the Fano resonance in PCS with different hole radiuses.

The single-layer Si PCS consists of square lattice with period *a* = 1000 nm, and the thickness of the Si PCS *t* = 160 nm. The reflection spectra for the single-layer PCS with a hole radius of 100 nm and 200 nm under surface-normal incidence (*θ* = 0°) are plotted out in Figure 2a. Azimuth angle *φ* has no effect on the reflectance spectrum of the PCS at surface-normal incidence due to 90-degree rotational symmetry [44]. The spectral feature for the resonances becomes sharper when *r* reduces from 200 nm to 100 nm. The *Q* of the resonances, extracted from the Fano-fitting [41], is 10,940 and 1654 for *r* = 100 nm and *r* = 200 nm, respectively. The sensitivity was calculated with the equation *S* = ∆*λ*/∆*n* by tracking the resonance shift at two different refractive indices—*n* = 1.33 and *n* = 1.34—for the aqueous analyte solution. As shown in Figure 2b, the resonance *Q* factor increases with the decrease of the radius. On the other hand, the sensitivity *S* only decreases slightly from 860 nm/RIU to 815 nm/ RIU when the radius decreases from 300 nm to 10 nm.

**Figure 3:**
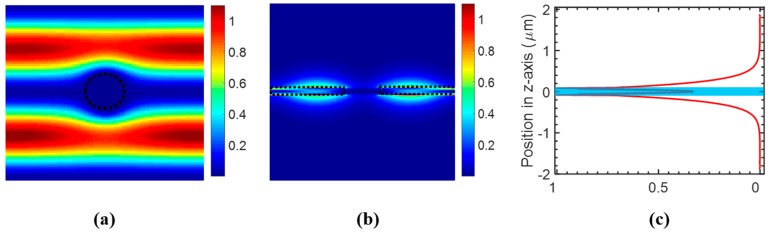
Simulated field distribution of the single layer PCS with *a* = 1000 nm, *r* = 100 nm, and *t* = 160 nm, at surface-normal incidence (*θ* = 0°). (**a**) *ε*|*E*|^2^ distribution at the center of the PCS in the *x*–*y* plane, with boundary of the hole labeled in dashed circle. (**b**) *ε*|*E*|^2^ distribution at the center of the hole in the *y*–*z* plane, with boundary of the Si PCS labeled in dashed rectangles. (**c**) Distribution of integrated *ε*|*E*|^2^ in one unit cell along vertical (*z*-axis) direction, with blue shaded area indicating the Si PCS.

The energy distribution *ε*|*E*|^2^ of the field at resonance wavelength was computed to estimate the optical integral *f* in the analyte region. Figure 3a shows the *ε*|*E*|^2^ distribution at the center of the Si PCS (*z* = 0) in the *x*–*y* plane, which shows almost no field distribution in the hole region. The *ε*|*E*|^2^ distribution at the center of the hole in the *y*–*z* plane is shown in Figure 3b. To calculate the amount of field energy in the analyte region, we integrated *ε*|*E*|^2^ in one unit cell along the *z* axis, which is plotted in Figure 3c. The blue shaded area is the Si PCS region. As analyte exists above and below the PCS, the calculated optical integral *f* in analyte is 0.733. The sensitivity is estimated to be 839 nm/RIU from Equation (2), which agrees well with the sensitivity of 830 nm/RIU calculated from resonance spectral tracking using S^4^.

#### 3.1.2. Case S_2_: Near Surface-Normal Incidence

**Figure 4:**
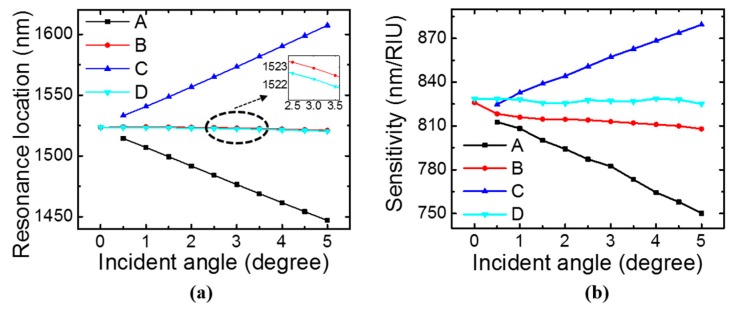
Simulation of single-layer PCS with *a* = 1000 nm, *r* = 100 nm, and *t* = 160 nm at different incident angles with *φ* = 0°. (**a**) Resonance spectral location dependence on incident angle. (**b**) Bulk sensitivity dependence on incident angle.

If the incident angle *θ* of the beam is not strictly zero, the symmetry is broken. At Γ point (***k*** = 0, *θ* = 0°), square lattice possesses a symmetry group of C_4υ_. If it moves from Γ point to X point (*θ* ≠ 0°, *φ* = 0°), the symmetry group changes to C_1h_ [45]. As shown in the inset of Figure 4a, the mode existing at *θ* = 0° splits into two modes, *B* and *D*. Two new modes *A* and *C* emerge in the spectra when *θ* is non-zero. They are symmetry-protected BIC at *θ* = 0° because there is a symmetry mismatch between them and the radiation modes [23,33]. Modes *A*, *B*, and *C* can be excited by source with TM polarization, while mode *D* can be excited by a source with TE polarization. Sensitivity of the four modes are plotted in Figure 4b. Mode *A* has lower sensitivity at larger angles, while mode *C* has higher sensitivity at larger angles. Sensitivity for modes *B* and *D* remains relatively unchanged in the incident angle range of 0–5°.

**Figure 5:**
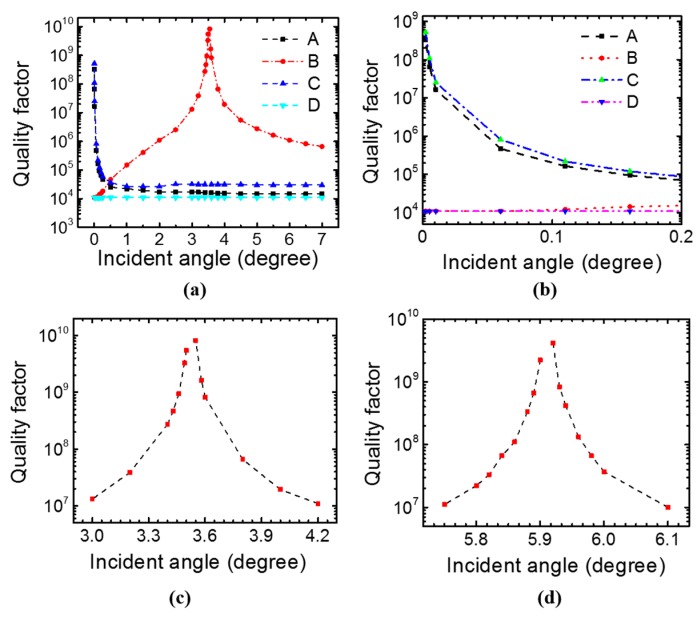
Study of quality factor in single-layer PCS at different incident angles with *φ* = 0°. Design of the PCS: *a* = 1000 nm, *t* = 160 nm, *r* = 100 nm in (**a**)–(**c**), and *r* = 200 nm in (**d**). *Q* factor of the four modes in PCS at an incident angle in the range of 0–7° (**a**) and at near-zero incident angles (**b**). Mode *B* at incident angles near infinite *Q* condition for the PCS with two different radii: *r* = 100 nm in (**c**) and *r* = 200 nm in (**d**), respectively.

Figure 5a shows the calculated quality factor with Fano fitting of the four modes at φ = 0° from the simulated reflection spectra. Modes *B* and *D* have a *Q* around 10^4^ at *θ* ≈ 0°, while the singly degenerate modes *A* and *C* have infinite *Q* when *θ* ≈ 0°. Figure 5b shows a zoom-in view of the *Q* for the four modes at small incident angles. Modes *A* and *C* have *Q* > 10^7^ in the incident angle range of 0–0.01°. When the azimuth angle *φ* is not zero, modes *A* and *C* occur at slightly different wavelengths in the reflection spectrum, but the quality factors for them are not affected much by the azimuth angle.

#### 3.1.3. Case S_3_: Specific Incident Angle

As we can see from Figure 5c, mode *B* has an extremely high *Q* at around 3.52°, where it becomes a quasi-bound state. Unlike the symmetry-protected BIC for mode *A* and *C* at ***k*** ≈ 0, the high-*Q* resonance for mode *B* is due to the destructive interference between the leakage channels above and below the PCS [32]. Mode *B* can achieve *Q* > 10^7^ in the incident angle range of 3°–4.2° when *φ* = 0°. When the radius *r* increases from 100 nm to 200 nm, the bound state for mode *B* occurs at an incident angle ~5.91°, as shown in Figure 5d. The results suggest that the BIC is robust. Small variations in device parameters (such as radius of the hole) only change the bound state to a different ***k*** value (or incident angle in this case).

**Figure 6:**
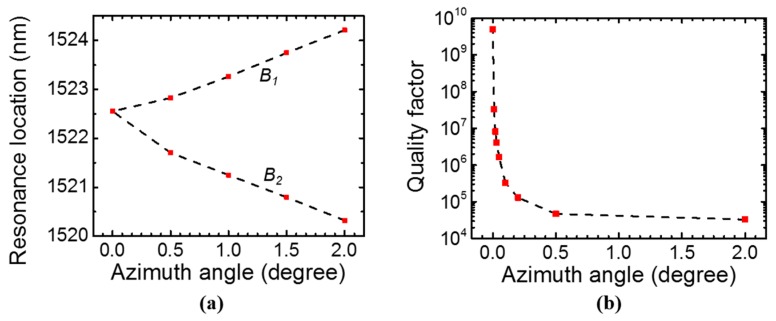
Simulation of mode *B* in single-layer PCS with *a* = 1000 nm, *r* = 100 nm, and *t* = 160 nm at *θ* = 3.5°. (**a**) Resonance spectral location dependence on azimuth angle *φ*. (**b**) Quality factor dependence of mode *B*_1_ on azimuth angle *φ*.

Unlike modes *A* and *C*, mode *B* splits to two modes *B*_1_ and *B*_2_ when the azimuth angle *φ* is not zero, as shown in Figure 6a. The quality factor reduces for mode *B*_1_ when azimuth angle increases, as indicated in Figure 6b. Mode *B*_2_ has lower *Q* than *B*_1_ and it is not discussed here. For mode *B*_1_ to achieve *Q* > 10^7^, the azimuth angle *φ* needs to be in the range of 0°–0.02°.

### 3.2. Coupled Bi-Layer PCS

**Figure 7:**
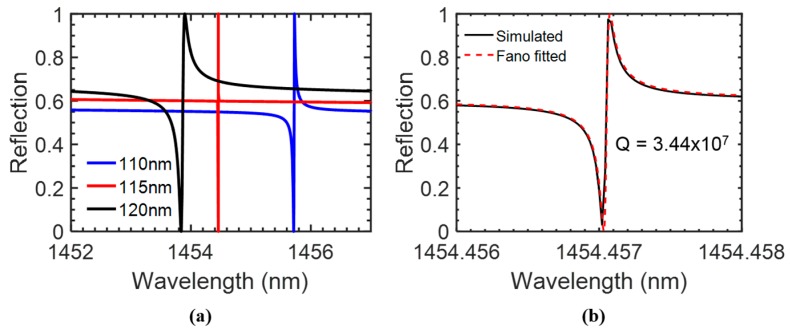
(**a**) Simulated reflection spectra of the coupled bi-layer PCS, with *a* = 1000 nm, *t*_1_ = *t*_2_ = 80 nm, *r* = 100 nm and three different gap distances, *t_g_*, at surface-normal incidence. (**b**) Zoom-in of the reflection spectrum of PCS with *t_g_* = 115 nm. Fano-fitting shows a *Q* factor of 3.44 × 10^7^.

The schematic of the coupled bi-layer PCS is shown in Figure 1b. The coupled bi-layer PCS is immersed in aqueous solution with RI of 1.33. The 2D Si PCS has a square lattice with period *a* = 1000 nm and a hole radius *r* = 100 nm. The thickness of top Si PCS *t*_1_ and bottom Si PCS *t*_2_ are both 80 nm. The gap distance *t_g_* affects the mode coupling between the top and bottom PCS [36]. We simulated the bi-layer PCS structure under surface-normal incidence condition with S^4^ software. The reflection spectra for the bi-layer PCS with gap distances *t_g_* of 110, 115, and 120 nm are plotted out in Figure 7a. Fano-fitting of the reflection spectrum for the coupled bi-layer PCS with *t_g_* = 115 nm shows a quality factor of 3.44 × 10^7^, as shown in Figure 7b.

**Figure 8:**
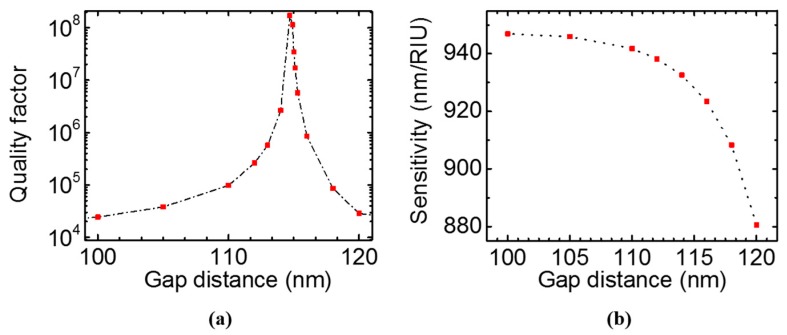
Quality factor (**a**) and sensitivity (**b**) for the resonance in bi-layer PCS of *a* = 1000 nm, *t*_1_ = *t*_2_ = 80 nm, *r* = 100 nm at surface-normal incidence, with gap distance, *t_g_*, varying from 100 nm to 120 nm.

We further studied quality factor of the Fano resonance dependence on the bi-layer gap distance in Figure 8a. The Fabry–Pérot BIC forms at gap distance *t_g_* around 114.7 nm. The top and bottom PCS act as a pair of perfect mirrors that trap waves between them. The bound state has an infinite *Q* and it cannot be observed in the spectrum. When the gap distance is slightly off, the bound state is perturbed, and it can couple to the external radiation mode with extremely high *Q*. The bulk sensitivity decreases from 950 nm/RIU to 880 nm/RIU when the gap distance increases from 100 nm to 120 nm, as shown in Figure 8b.

**Figure 9:**
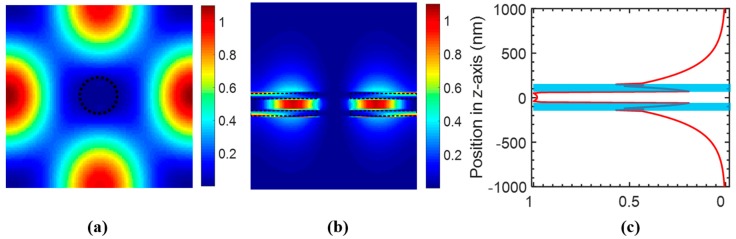
Simulation of field distribution of bi-layer PCS with *a* = 1000 nm, *t*_1_ = *t*_2_ = 80 nm, *r* = 100 nm, *t_g_* = 115 nm at surface-normal incidence. (**a**) *ε|E|*^2^ distribution at the center of the top PCS in the *x*–*y* plane, with hole boundary shown in the dashed circle. (**b**) *ε|E|*^2^ distribution at the center of the hole in the *y*–*z* plane, dashed rectangles show the boundary of top and bottom Si PCS. (**c**) Distribution of integrated *ε|E|*^2^ in one unit cell along vertical (*z*-axis) direction, with the blue shaded area showing the top and bottom Si PCS.

We computed the field distribution for the coupled bi-layer PCS with *t_g_* = 115 nm using FDTD method. Figure 9a shows the electric field energy profile at the center of the top PCS (*z* = 100 nm) in the *x*–*y* plane. The field energy distribution at the center of the hole in the *y*–*z* plane is shown in Figure 9b. The field is largely concentrated in the gap region between the top and bottom PCS, with large extension to the media above and below the slab. The integrated field energy in one unit cell is plotted in Figure 9c. The optical overlap integral *f* in the gap analyte region is around 0.33, while *f* is around 0.26 in the analyte region above the top PCS or beneath the bottom PCS. The total optical overlap integral *f* in the analyte outside the Si PCS is around 0.85. The sensitivity could be estimated to be 930 nm/RIU based on Equation (2). The sensitivity calculated from spectral resonance tracking is around 925 nm/RIU, which agrees with the calculated sensitivity from optical field overlap integral *f*.

## 4. Experimental Results

**Figure 10:**
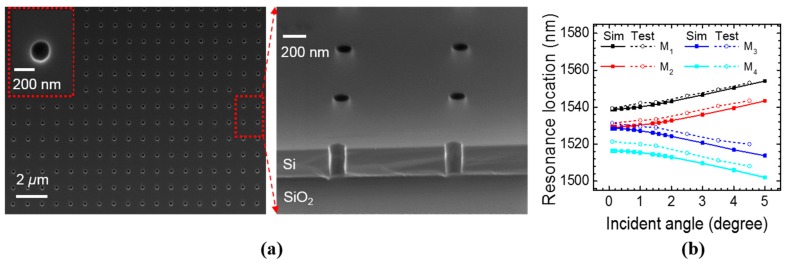
(**a**) SEM top view and angled cross-sectional view of one single layer PCS on SOI substrate, with a zoom-in image of one air hole in the inset. The PCS parameters: *a* = 990 nm, *r* = 85 nm, and *t* = 250 nm. (**b**) Simulated and tested resonance locations for the four modes of the PCS on SOI substrate at different incident angles. Simulated data points are represented by squares and connected by solid lines, and tested data points are represented by circles and connected by dashed lines.

As a proof-of-concept to the theoretical studies, we experimentally demonstrate one single-layer PCS design, which represents the Case S_2_ (near surface-normal incidence case) discussed in Section 3. We fabricated the single layer PCS on silicon on insulator (SOI) substrate with electron beam lithography (EBL) and dry etching with HBr/Cl_2_ gases. The top Si device layer thickness is 250 nm and the buried oxide thickness is 3 μm. The top view and cross-sectional view scanning electron microscope (SEM) images are shown in Figure 10a. The device was characterized with the cross-polarization technique as detailed in a previous work [23]. The measured and simulated resonance locations of the four modes at different incident angle from 0.1° to 5° are plotted in Figure 10b. The measured results agree well with the simulation results. 

**Figure 11:**
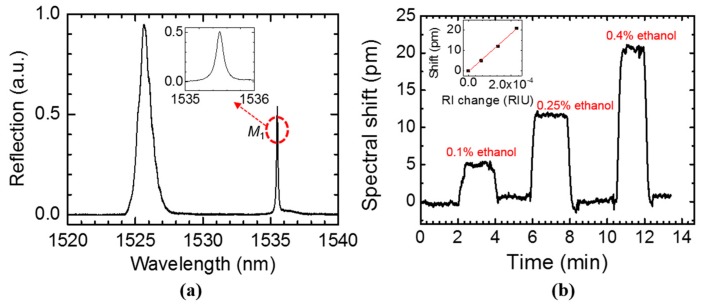
(**a**) Measured reflection spectrum of one single layer PCS immersed in water, with cross-polarizer technique. A zoom-in spectrum of mode *M*_1_ is shown in the inset. The PCS parameters: *a* = 970 nm, *r* = 85 nm, and *t* = 250 nm. (**b**) Sensorgram for injecting different concentrations of ethanol/DI water mixture and rinsing with water. The inset shows spectral shift as a function of refractive index change, and the sensitivity of the PCS is fitted to be 94 nm/RIU.

The reflection spectrum of single layer PCS immersed in water at near surface-normal incidence (Case S_2_) is shown in Figure 11a. The measured quality factor of mode 1 (*M*_1_) is 1.2 × 10^4^ obtained from Lorentzian fitting, which is limited by water absorption in the 1400–1600 nm region (imaginary part of the refractive index of water, *n*_i_, is around 10^−4^) [46]. The simulation in Section 3 only considers the radiation loss (*Q*_rad_) in the structure, while neglecting the material absorption loss and the scattering loss. If the scattering loss is negligible in experiment, the total loss can be related to the radiation loss and material loss by the following equation 1/*Q*_total_ = 1/*Q*_rad_ + 1/*Q*_abs_ [47]. Absorption of Si is negligible in the 1400–1600 nm range, and thus *Q*_abs_ is mainly due to water absorption and it can be calculated as *Q*_abs_ = *n*_r_/2*fn*_i_, where *n*_r_ is the real part of the refractive index of water (*n*_r_ = 1.33 at 1400–1600 nm), and *f* is the optical overlap integral as defined in Equation (1), which is around 0.11 for the measured single layer PCS. *Q*_abs_ can be estimated to be 6 × 10^4^ which is the upper bound for the *Q*_total_. 

Ethanol/deionized (DI) water mixture with different concentrations (0.1–0.4% volume ratio) were injected into the polydimethylsiloxane (PDMS) microfluidic channel bonded on the device to test the resonance shift of mode *M*_1_. The sensorgram of mode *M*_1_ with 0.1%, 0.25%, and 0.4% ethanol/DI water mixture are shown in Figure 11b. DI water was used to rinse the PDMS chamber after each test run of ethanol/DI water mixture. In the inset of Figure 11b, the spectral shift of mode *M*_1_ is plotted with respect to RI change of the analyte above the PCS surface. The 0.1% ethanol/DI water mixture has a 6 × 10^−5^ RIU RI change compared to DI water and induced around 5 pm spectral shift, which can be clearly distinguished. The bulk sensitivity is 94 nm/RIU obtained from the linear fitting of the measured results. The sensitivity could be increased by decreasing the PCS thickness and suspending the single layer PCS in the analyte solution so that a larger optical field will interact with the analyte. As discussed in Section 3, the sensitivity for single layer PCS can be as high as 800 nm/RIU with the optimized design.

## 5. Discussion

All the four cases discussed in this work can achieve high *Q* and *S* simultaneously. Detection limit can be estimated from *Q* and *S*. Spectral linewidth *δλ* is related to resonance wavelength *λ*_0_ and *Q* by *δλ* = *λ*_0_/*Q*. Assume a spectral resolution *R*’ as 1/20th of the spectral linewidth [2,48], we can achieve *DL* = *R*’*/S*~10^−8^ RIU, with *Q* = 10^7^ and *S* = 800 nm/RIU at *λ*_0_~1550 nm. The requirements in each configuration studied in this work to achieve *Q* = 10^7^ and *S* = 800 nm/RIU are summarized in Table 1. For single layer PCS at surface-normal incidence (case S_1_), the radius of the hole needs to be less than 15 nm to have *DL* < 10^−8^ RIU. For coupled bi-layer PCS to achieve *DL* < 10^−8^ RIU at surface-normal incidence, the gap distance *t_g_* is required to be in less than 0.5 nm away from 114.7 nm. Small hole size or high accuracy in gap distance and PCS thickness control are challenging in terms of fabrication of the device. For single layer PCS with oblique incident light (case S_2_ and S_3_), the hole size can be relatively large. *DL* < 10^−8^ RIU can be achieved for hole radius of 100–200 nm by controlling the incident angle and the azimuth angle. Case S_3_ has larger incident angle tolerance than case S_2_. It can maintain high *Q* > 10^7^ in an incident angle range of 3°–4.2° with *φ* = 0°. The larger incident angle range to maintain high *Q* is beneficial in relaxing the optical alignment requirement in practical sensor systems, as typically the incident Gaussian beam consists of a spread of ***k*** points (or incident angle) related to the lateral size of the beam [32]. However, case S_3_ has less tolerance in terms of azimuth angle compared to case S_2_. In case S_2_, quality factors of mode *A* and *C* are not affected much when azimuth angle *φ* is not zero, while *φ* needs to be less than 0.02° for mode *B* in case S_3_ to achieve *Q* > 10^7^. In experiment, the azimuth angle can be controlled by rotating the sample stage or adjusting the principle axis of the linear polarizer to control the polarization direction of the incident beam.

If we tune the incident angle for coupled bi-layer PCS, the dependence of *Q* on incident angle for the four modes shows a similar trend as that for the single-layer PCS. For one coupled bi-layer PCS design with *t*_1_ = *t*_2_ = *t_g_* = 80 nm, *a* = 1000 nm, and *r* = 100 nm, mode *A* and *C* have extremely high *Q* at *θ* ~0°, while mode *B* can achieve near infinite *Q* at an incident angle of ~3°. The result suggests that the approaches to achieve high *Q* in single layer PCS by tuning incident angle also apply to coupled bi-layer PCS.

In biochemical sensing experiments, aqueous solution has a large absorption in the 1400–1600 nm range. For the two proposed PCS structures in Section 3, single layer PCS and coupled bi-layer PCS, high sensitivity is attributed to the large optical overlap integral with the analyte. However, a large optical overlap integral results in higher absorption loss, which limits the ultimate Q factor in experiment through *Q*_abs_, although *Q*_rad_ can go up to 10^7^ for both cases. Therefore, *Q* and *S* are trade-offs in biochemical sensors in the 1400–1600 nm region. To fully take advantage of the extremely high *Q* of the silicon-based PCS, aqueous solution with lower absorption loss can be used, such as deuterium oxide [49]. Nevertheless, the principles discussed in this work are applicable to PCS structures fabricated with other materials, such as Si_3_N_4_ and TiO_2_, for biochemical sensing in the visible wavelength region (400–800 nm), where water absorption is three orders of magnitude lower than that in the 1400–1600 nm region.

## 6. Conclusions

In this work, we have presented the designs of single-layer and coupled bi-layer PCS based on BIC to achieve high *Q* and high *S* at the same time. A theoretical high *Q* of 10^7^ and *S* of 800 nm/RIU (*DL*~10^−8^ RIU) were achieved by tuning the hole radius, the incident angle, and azimuth angle of the light for single-layer PCS. In the case of coupled bi-layer PCS, the separation distance between the top and the bottom PCS is varied and investigated to achieve high *Q* and *S*. Trade-offs of different designs are investigated thoroughly for practical implementation. One non-optimal design of single-layer PCS was fabricated and characterized as a proof-of-concept, where we demonstrated a sensitivity of 94 nm/RIU and a quality factor of 1.2 × 10^4^, with a detection limit of 6 × 10^−5^ RIU. The sensing performance of the BIC-based PhC resonators is superior to traditional PhC designs because of lower radiation loss and stronger light interaction between the mode and the analyte. The compact defect-free PCS sensor platform with low detection limit provides new approaches to real-time and on-chip biosensing with microfluidics integration. 

## Figures and Tables

**Table 1:** Summary of different approaches to achieve *Q* > 10^7^, *S* > 800 nm/RIU and *DL* < 10^−8^ RIU in PCS sensor design.

Configuration	Cases	Incident angle *θ*	Requirement
Single layer	S_1_	0°	Radius *r* < 15 nm
Single layer	S_2_	≈0°	0° < *θ* < 0.01°
Single layer	S_3_	>0°	3° < *θ* < 4.2°; φ < 0.02°
Bi-layer	-	0°	Gap *t_g_* = 114.2–115.2 nm
